# Evenness is important in assessing progress towards sustainable development goals

**DOI:** 10.1093/nsr/nwaa238

**Published:** 2020-09-28

**Authors:** Yali Liu, Jianqing Du, Yanfen Wang, Xiaoyong Cui, Jichang Dong, Yanbin Hao, Kai Xue, Hongbo Duan, Anquan Xia, Yi Hu, Zhi Dong, Bingfang Wu, Xinquan Zhao, Bojie Fu

**Affiliations:** College of Resources and Environment, University of Chinese Academy of Sciences, Beijing 100049, China; College of Life Sciences, University of Chinese Academy of Sciences, Beijing 100049, China; College of Resources and Environment, University of Chinese Academy of Sciences, Beijing 100049, China; CAS Center for Excellence in Tibetan Plateau Earth Sciences, Chinese Academy of Sciences, Beijing 100101, China; Yanshan Earth Critical Zone and Surface Fluxes Research Station, University of Chinese Academy of Sciences, Beijing 100049, China; College of Life Sciences, University of Chinese Academy of Sciences, Beijing 100049, China; CAS Center for Excellence in Tibetan Plateau Earth Sciences, Chinese Academy of Sciences, Beijing 100101, China; Yanshan Earth Critical Zone and Surface Fluxes Research Station, University of Chinese Academy of Sciences, Beijing 100049, China; School of Economics & Management, University of Chinese Academy of Sciences, Beijing 100190, China; College of Life Sciences, University of Chinese Academy of Sciences, Beijing 100049, China; CAS Center for Excellence in Tibetan Plateau Earth Sciences, Chinese Academy of Sciences, Beijing 100101, China; Yanshan Earth Critical Zone and Surface Fluxes Research Station, University of Chinese Academy of Sciences, Beijing 100049, China; College of Resources and Environment, University of Chinese Academy of Sciences, Beijing 100049, China; CAS Center for Excellence in Tibetan Plateau Earth Sciences, Chinese Academy of Sciences, Beijing 100101, China; Yanshan Earth Critical Zone and Surface Fluxes Research Station, University of Chinese Academy of Sciences, Beijing 100049, China; School of Economics & Management, University of Chinese Academy of Sciences, Beijing 100190, China; College of Life Sciences, University of Chinese Academy of Sciences, Beijing 100049, China; School of Economics & Management, University of Chinese Academy of Sciences, Beijing 100190, China; School of Innovation and Entrepreneurship, University of Chinese Academy of Sciences, Beijing 100190, China; College of Resources and Environment, University of Chinese Academy of Sciences, Beijing 100049, China; State Key Laboratory of Remote Sensing Science, Aerospace Information Research Institute, Chinese Academy of Sciences, Beijing 100101, China; Key Laboratory of Adaptation and Evolution of Plateau Biota, Northwest Institute of Plateau Biology, Chinese Academy of Sciences, Xining 810008, China; College of Resources and Environment, University of Chinese Academy of Sciences, Beijing 100049, China; State Key Laboratory of Urban and Regional Ecology, Research Center for Eco-Environmental Sciences, Chinese Academy of Sciences, Beijing 100085, China

**Keywords:** sustainable development goals, evenness, development pathway, adaptive strategy, China

## Abstract

Sustainable development goals (SDGs) emphasize a holistic achievement instead of cherry-picking a few. However, no assessment has quantitatively considered the evenness among all 17 goals. Here, we propose a systematic method, which first integrates both the evenness and the overall status of all goals, to distinguish the ideal development pathways from the uneven ones and then revisit the development trajectory in China from 2000 to 2015. Our results suggest that, despite the remarkable progress, a bottleneck has occurred in China since 2013 due to the stagnant developments in some SDGs. However, many far-reaching policies in China have been targeting these deficiencies since then, providing a perspective on how a country approaches sustainable development by promoting evenness among all SDGs. Our results also indicate that regions with the slowest progress are the developed provinces, owing to the persistent uneven status of all goals. Our study demonstrates the importance of adopting evenness in assessing and guiding sustainable development.

## INTRODUCTION

Since sustainable development goals (SDGs) were addressed in 2015 [1], many studies have explored different methods to assess all 17 goals to understand the progress and to guide policy implementation towards SDGs [2–5]. Although most studies have suggested that all 17 SDGs should be equally addressed [2–4], still no current assessment index has quantitatively included the developing evenness of all 17 SDGs. As a widely used index in ecology [6], the concept of ‘evenness’ originated as a supplementary of species richness in the measurements of biodiversity, which describes the distribution of relative abundance among species. And a large number of species with equal distributed relative abundance is considered as ‘high biodiversity’. Analogously, evenness can be used to investigate the differences in the performance among 17 SDGs. It is important since many SDGs interact with one another [7–9], while negative interactions might result in unevenness among all SDGs and impede the holistic achievement of SDGs [10]. For instance, fast economic development at the cost of the environment is uneven and unsustainable (e.g. a 100% performance on SDG 8, economic growth, but a zero performance on SDG 15, life on land), while simultaneous achievement in economic development and environmental protection is even (e.g. 50% performance on both SDG 8 and 15). However, these two types of development could be concluded with similar performances (50%, if all other SDGs also have a 50% performance) based on assessment only comparing the average performance of all SDGs. Therefore, it is necessary to integrate evenness into SDG assessment to keep a country from over-optimism and help it to implement more deficiency-dependent strategies towards SDGs.

**Figure 1.**
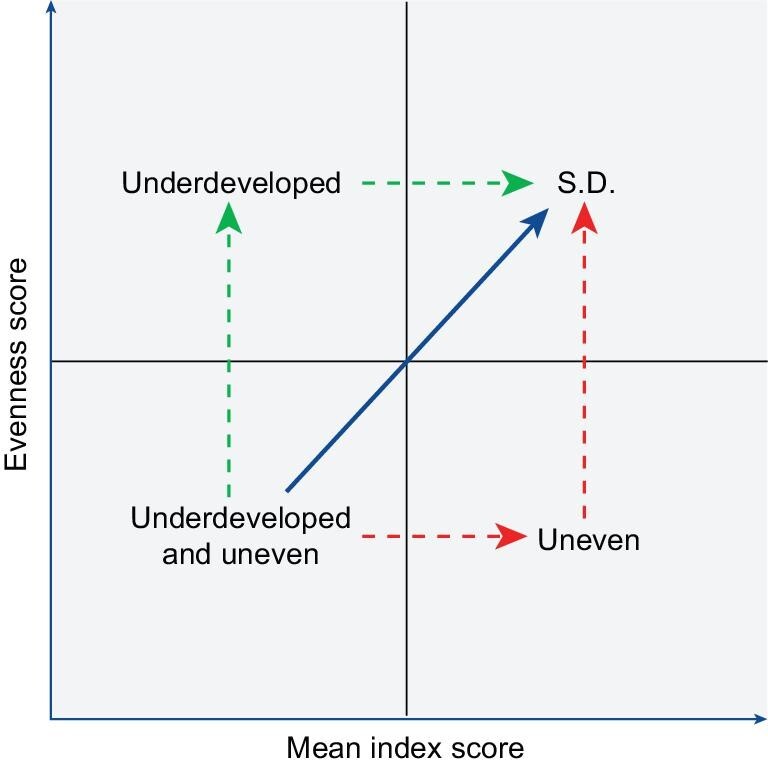
Pathways approaching sustainable development by adopting evenness. All statuses refer to relative statuses. S.D. stands for the relative ideal status towards SDGs.

Moreover, integrating evenness might also help governments to match adaptive strategies to places. As shown in Fig. 1, regions in different developing stages need different strategies towards SDGs. Relatively uneven regions may need more deficiency-dependent strategies and relatively underdeveloped regions may need more support from the central government, while these regions can collaborate in a mutually beneficial way. For instance, economically developed regions with a stressful environment are uneven, whereas they can benefit from their adjacent underdeveloped regions with a well-protected environment, such as clean water. In turn, economically developed regions can provide substantial support through ecological compensations, to encourage the underdeveloped regions to take sustainable economic development with the least cost for the environment. As for the underdeveloped and uneven regions, there are three potential pathways (Fig. 1): (i) the ideal is the blue pathway which achieves all 17 SDGs simultaneously; (ii) the green pathway starts with making up for the deficiency; (iii) the red pathway can be unsustainable when the development is at the cost of some SDGs. Therefore, evenness is crucial for adaptive strategies approaching SDGs at both national and regional levels.

Since the economic reform in 1978, China has experienced rapid economic development. In the meantime, many studies have suggested that China’s development is unsustainable, especially at the cost of the environment [11], with regard to unsustainable energy consumption [12,13], groundwater depletion [14,15], water and air pollution [16,17], and biodiversity decline [18,19]. Although a recent study indicated that a notable improvement towards SDGs had been made in China from 2000 to 2015 [2], it is still not sufficient to demonstrate whether or not China has been on the right track towards sustainable development if evenness among all SDGs was not considered. Therefore, the present study is to address the following two questions: (i) what pathway had China experienced from 2000 to 2015 when considering the evenness of 17 SDGs? (ii) What lessons can be learned from integrating evenness in assessing sustainable development at national and regional levels?

We used a radar chart method [20] to quantify the evenness score (ES) of all SDGs over space and time based on the SDG Index scores of China, which were obtained from a previous study [2]. We developed the sustainable development score (SDS) to re-evaluate China's development from 2000–2015 at both national and regional levels, and compare the performance between economically developed and developing provinces (see Methods), by integrating both the evenness score from the present study and the quoted SDG Index score (referring to the mean index score in the present study, MIS). Then, we explored the developing pathway at both national and regional levels and the existing challenges towards SDGs in China.

## RESULTS AND DISCUSSION

### China's progress towards SDGs by improving evenness score at the national level

**Figure 2.**
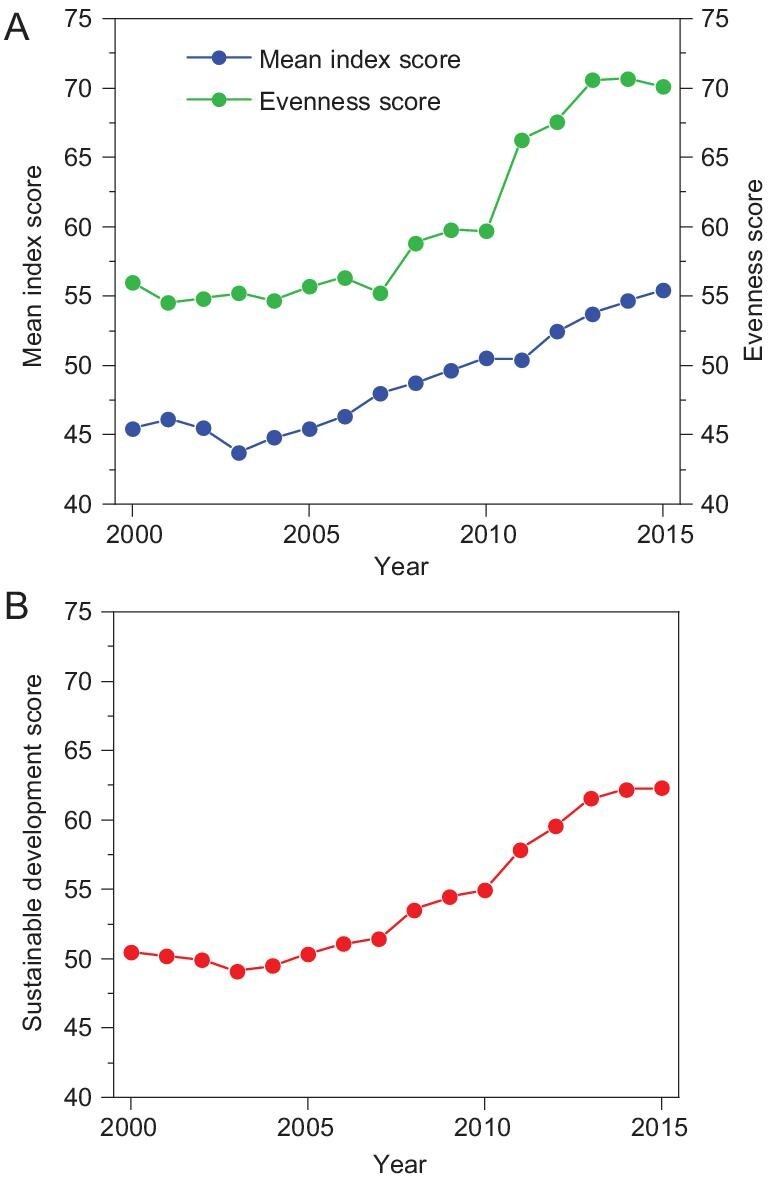
Changes in (A) China's SDG mean index score and evenness score, and (B) sustainable development score.

Both the mean index score and evenness score increased by over 20% in China from 2000 to 2015 (Fig. 2A). Based on these results, China's development since the 21st century might have inadequacies as previous studies suggested [13,14,19,21]. However, remarkable progress has been made by China towards SDGs, not only in the increase of overall performance of all 17 SDGs [2], but also the noticeable endeavors in balancing them.

Inconsistent with the stable increasing trend of the mean index score since 2003, the evenness score was likely to be stepped up during the study period (Fig. 2A). Two increasing periods were identified as 2007–2008 and 2010–2013. A similar trend was also found for the sustainable development score, which had stagnated since 2013 (Fig. 2B). Many underdeveloped SDGs had been largely improved in two periods, namely, 2000–2008 and 2008–2015 (Fig. S1). For instance, the scores for SDG 9, 15 and 17 had increased by 48%, 28% and 118%, respectively, from 2000 to 2008; the scores for SDG 1, 9 and 10 had increased by 55%, 46% and 93%, respectively, from 2008 to 2015. These two dramatic increases in evenness were associated with two important periods in China, the Beijing Olympic Games in 2008 and the new term of the central government since 2012. Numerous efforts had been taken by the Chinese government to improve the deficient SDGs during these two periods, making China more open, less unequal and more environmentally friendly. After the Beijing Olympic Games, many previously underdeveloped SDGs were significantly improved. Among them, major progress was found on SDG 17 (partnerships for the goals). The total export–import volume and foreign investments, indicators of SDG 17, had increased by 358% and 60% respectively from 2000 to 2008, with a dramatic increase of foreign investments by 22% in 2008 compared with those in the previous year [22]. These increases demonstrated the Chinese government’s resolution in expanding international cooperation. The increasing international trade further promoted the economic development of China [2], which is indicated through the improvement of SDG 8 (decent work and economic growth). Other intensive actions were also conducted to approach SDG 7 (affordable and clean energy) [23,24]. After 2008, China's challenges towards SDGs were mainly concerned with SDG 10 (reduced inequality), 9 (industry, innovation and infrastructure), 14 (life below water), 15 (life on land), 17 (partnerships for the goals) and 1 (no poverty), starting from the lowest SDG score (Fig. S1). Nevertheless, many endeavors had been made to reduce the unevenness among the 17 SDGs since 2008, particularly by the new term of the central government since 2012. Initiatives and policies, such as ‘China Rural Poverty Alleviation and Development Program (2011–2020)’ and later the Targeted Poverty Alleviation Strategy in 2013, had been conducted to reduce poverty and eliminate economic inequality [25]. ‘China Biodiversity Conservation Strategy and Action Plan (2011–2030)’ was proposed in 2010 to improve SDGs 14 and 15 [26]. Moreover, since 2012, the Chinese government had paid unprecedented attention to ecological conservation, by enacting a series of action plans for the prevention and control of air pollution, water pollution and soil pollution in 2013, 2015 and 2016, respectively. With all these efforts, China's economic growth has decoupled from environmental impacts and energy consumptions since 2015 [27,28]. Overall, China exhibited a quite desirable pathway towards all SDGs at the national level, contradicting previous conclusions that China’s development focused too much on the economy and therefore was unsustainable [11,29]. This suggests a transformation of China's policies towards sustainable development since 2000.

### China's progress towards SDGs by improving evenness score at the regional level

**Figure 3.**
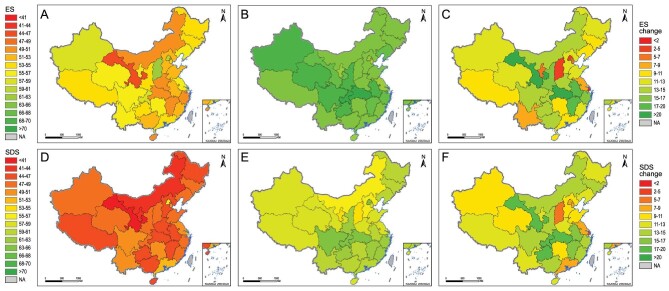
The spatial pattern of evenness score (ES) and sustainable development score (SDS) in 2000 and 2015, and corresponding changes from 2000 to 2015. (A) and (B) ES in 2000 and 2015, respectively; (C) changes of ES; (D) and (E) SDS in 2000 and 2015, respectively; (F) changes of SDS. NA, not available.

At the regional level, all provinces had increased evenness score and sustainable development score from 2000 to 2015 (Fig. 3; Table S1). The improvement in provincial evenness scores ranged from 2.94% (Shanghai) to 46.2% (Gansu) from 2000 to 2015, while the improvement in provincial sustainable development scores ranged from a 6.95% increase (Shanghai) to a 43.69% increase (Gansu) from 2000 to 2015. Notably, provinces in northern China had the highest average evenness score in 2000 and the lowest in 2015 (Table S1), and also the least improvement in the sustainable development score from 2000 to 2015 (Fig. 3F; Table S1), suggesting an uneven developing pathway that needs attention from local governments. These results indicated a regional unevenness towards sustainable development among 31 provinces; however, such regional unevenness had decreased from 7% in 2000 to 4% in 2015 (referring to the coefficient of variation of sustainable development score among 31 provinces). Moreover, most SDGs exhibited an increasing evenness among all provinces from 2000 to 2015 (except for SDG 12, responsible consumption and production, and SDG 15, life on land; Fig. S2; Table S2), suggesting a declining inequality of the performance of most SDGs among provinces. Notably, many uneven SDGs among provinces in 2000 had been largely improved during these 15 years (e.g. SDG 10, reduced inequalities). In 2015, the three most even SDGs among 31 provinces were SDG 3 (good health and well-being), SDG 13 (climate action) and SDG 2 (zero hunger), whereas the three most uneven SDGs among 31 provinces were SDG 7 (affordable and clean energy), SDG 9 (industry, innovation and infrastructure) and SDG 14 (life below water) (Table S2). These results indicated that holistic action plans at the national level on the adjustment of energy structure and industrial structure, along with the protection of water environment, are urgent in achieving SDGs [13,19].

### Developing pathways and the bottleneck towards SDGs at national and regional levels

Our analyses visualized the footprint of China's development from 2000 to 2015. The ideal pathway is defined as one which simultaneously achieves the improvement of all 17 SDGs. In this regard, the developing pathway is classified into five degrees: slightly uneven, uneven, slightly underdeveloped, underdeveloped, and relatively ideal, based on the angle between the actual pathway and the ideal pathway (see Methods). At the national level, significant progress was found from 2010 to 2015 (Fig. S3). Overall, China presented a quite even but slightly underdeveloped pathway (θ = 54.8°). However, after 15 years’ development, a bottleneck seemed to

occur in China (Fig. 2B). Although China's performance towards all 17 SDGs was relatively even in 2015, there were still a few poorly achieved SDGs, including SDG 8 (decent work and economic growth), 14 (life below water), 15 (life on land) and 17 (partnerships for the goals). SDG 8 was proved to be highly dependent on SDG 17 [2,29], whereas it was negatively correlated with SDG 14 and 15 [30], suggesting China was facing a dilemma of achieving both economic development and biodiversity conservation. Additionally, China had been stuck in promoting international collaborations since 2008, indicating that the major impacts of the Beijing Olympic Games did not last as time passed.

**Figure 4.**
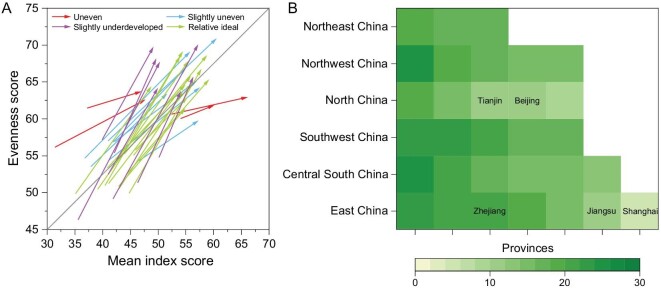
Developing pathways and effective development scores of 31 provinces from 2000 to 2015. (A) Vector diagram visualizing the developing pathways of 31 provinces (see Methods); (B) effective development scores (EDS) among provinces sorted by geographical locations. The gray diagonal in (A) marks the perfect pathway with a slope of 1, and the colored arrows visualize the pathways among provinces. The color depth in (B) refers to the EDS. The corresponding provinces in (B) refer to Table S4, meanwhile the top five developed provinces are marked accordingly.

At the provincial level, 13 out of 31 provinces had experienced a relatively ideal pathway with simultaneous improvement in both the evenness score and the mean index score (Fig. 4A; Table S3). Only four were considered as uneven (namely, Beijing, Shanxi, Shanghai and Ningxia). Others were either slightly uneven or slightly underdeveloped (Fig. 4A; Table S3).

The effective development scores (EDS, see Methods) were further used to quantify the progress of 31 provinces towards SDGs from 2000 to 2015 through assessing the developing pathway. Four of the top five economically developed regions had nearly no improvement from 2000 to 2015 (namely, Shanghai, Beijing, Tianjin and Jiangsu; Fig. 4B; Table S4). Additionally, the average evenness score for the economically developing provinces became higher than that of the economically developed provinces since 2010, and there was a significant increase of evenness in developing provinces, not in developed ones, between 2010 and 2015 (Fig. S4A; Table S5). Although economically developed provinces always had higher average sustainable development scores than economically developing provinces, their growth rate in sustainable development score was relatively lower (21.63% vs. 32.48%; Fig. S4B; Table S5). Similar patterns were also observed between the top five economically developed provinces and the bottom five economically developing provinces, whereas no significant increase in the sustainable development score was detected in the top five developed provinces from 2010 to 2015 (Fig. S4B). These situations were mainly ascribed to the persistent uneven status among all SDGs in the economically developed regions, particularly in Shanghai, Beijing, Tianjin and Jiangsu (Table S1). These results suggested that the economically developed regions are actually facing more challenges in approaching SDGs, probably due to the environmental stresses derived from their high population density. These provinces were also the top four provinces with the highest proportion of urban areas in China in 2015 [22], suggesting that the urbanization might have slowed down the sustainable development progress in economically developed regions. For example, both Beijing and Shanghai were lacking in progress in SDG 5 (gender equality), 6 (clean water and sanitation) and 15 (life on land) in 2015. During the past few decades, Beijing and Shanghai have experienced rapid urbanization [31,32], resulting in scarce natural habitats for life on land [22]. The population growth along with urbanization caused much higher water stress in Beijing and Shanghai compared with the rest of China [2,22]. Additionally, Beijing and Shanghai also had a much lower ratio of water footprint to crop yield production [22], an indicator for SDG 6 [2], owing to the reduction of arable land and grain yield resulting from urbanization [33]. All these environmental stresses resulting from urbanization had set an upper limit and left almost no room for the improvement of SDG 6 and 15. Therefore, the bottleneck for economically developed regions in approaching SDGs is mainly ascribed to the resource and environmental constraints derived from their high population density along with urbanization. Additionally, gender equality is another typical issue for developed regions, probably due to the financial pressures for households. For instance, the low maternal employment ratios in Beijing and Shanghai [34] probably relate to the high expense of child care [35], as some middle-class women stay at home to attend children.

### Current SDG status and policy implications

**Figure 5.**
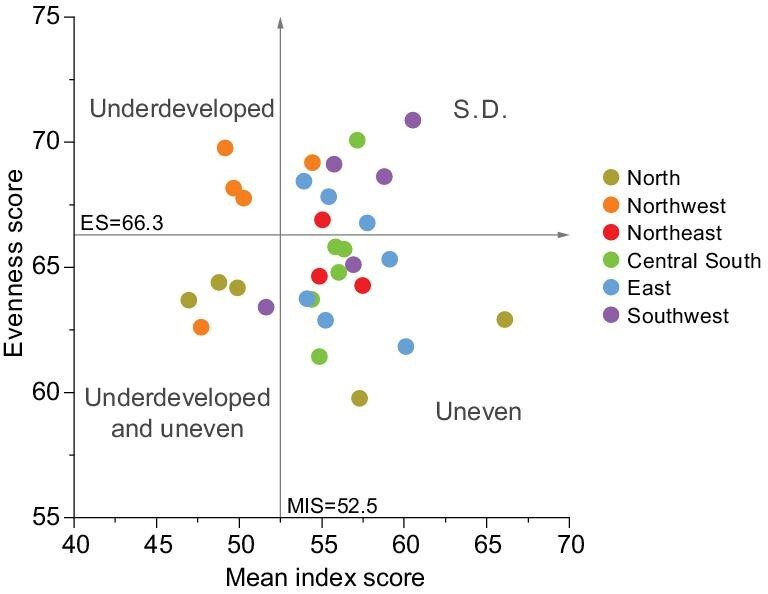
Regional differences among 31 provinces towards SDGs in 2015. The K-mean method is used to set up thresholds for ES and MIS to distinguish between relatively good and laggard status. All provinces are grouped by geographical locations and presented with different colors. S.D., relatively sustainably developed.

All 31 provinces were divided into four categories in terms of their relative SDG status in 2015 (see Methods). About half the provinces were still relatively uneven (although many were quite close to a relatively sustainably developed status, e.g. the northeast provinces and most of the central south provinces), while only three provinces were relatively underdeveloped; additionally, five were stuck in both (Fig. 5; Table S6). North and Northwest China still faced troubles on the route to sustainable development. Most northwest provinces were underdeveloped, while all northern provinces were either quite uneven, or uneven and underdeveloped (Fig. 5; Table S6). Therefore, regional integration and cooperation might be effective in holistically achieving SDGs across regions.

The relatively underdeveloped and uneven regions may collaborate in a complementary way towards SDGs, a method particularly beneficial for economically developed regions (mostly uneven). For instance, the stagnant progress in SDG 15 (life on land) in Beijing may be improved by collaborating with its surrounding regions (e.g. Hebei, which has much lower gross domestic product (GDP) per capita than Beijing) on ecological conservation. A feasible ecological compensation for Hebei province and the industrial relocation from Beijing to Hebei should help with the conservation and restoration of natural habitats in Beijing and its surrounding areas, and also help Hebei achieve economic development. Such regional cooperation between relatively uneven provinces (e.g. Beijing) and relatively underdeveloped provinces (e.g. Hebei) could be a chance to promote sustainable development in North China. Moreover, the water crisis in Beijing has been mitigated by the South-North Water Transfer Project which transfers water from the Hanjiang river basin to Beijing, where it has notably increased water resources during the last decade [14]. On the other hand, the Hanjiang river basin also benefits from this project through investment in ecological conservation and preservation as well as reasonable ecological compensation [36]. Such a collaboration is especially effective between underdeveloped regions with high gross ecosystem production (GEP) but low efficiency to convert it into GDP, and developed regions with low GEP but high efficiency to convert it into GDP, e.g. between Qinghai (underdeveloped) and Shanghai (uneven), both of which are linked by the Yangtze River. Such cooperation between upstream and downstream regions may help with sustainable development in Northwest China, located in the upstream of the Yangtze River and Yellow River.

## CONCLUSION

As a retrospective study, we have explored whether the Chinese government has taken action to respond to the raised issues in 2015. Luckily, many far-reaching policies have been targeting the four identified deficiencies in SDGs in China since 2015. For instance, the initiatives of ‘Mass Entrepreneurship and Innovation’ and ‘Made in China 2025’ aim at SDG 8, and the ecological civilization vision and relevant actions, such as the fishing ban in the Yangtze River from 2020 to 2030, target SDGs 14 and 15, and the Belt and Road initiative at SDG 17. All these efforts have demonstrated China's resolution towards an even development. By integrating all these findings, we are convinced that evenness expands the implications of sustainable development assessment and helps the government to match adaptive strategies to places. Moreover, the methods proposed in the present study can be applied to the rest of the world since they generally follow the widely adopted methods in data selection, normalization and calculation of SDG scores. Overall, our study provides a new perspective on how a country approaches sustainable development by promoting evenness among all SDGs.

## METHODS

### Data sources

All SDG scores (the score for each SDG) and SDG index scores (average of all 17 SDG scores) over time and space at national and regional levels were quoted from Xu *et al.* [2]. More details on indicator selection, data source and calculations can be found in that article. Briefly, 119 SDG indicators were selected in their calculations, with 3 to 18 for each SDG. The score for each SDG was calculated using the arithmetic mean of all corresponding SDG indicators’ normalized values. The SDG index score was the mean value of all 17 SDG scores. The methods for normalization and calculation were generally based on the 2018 SDG Index and Dashboards Report [4]. Moreover, they also explored the uncertainty introduced by the number of SDG indicators selected for calculating each SDG score and found that the median SDG score was almost constant when the number of selected indicators per SDG was over two. Therefore, the SDG scores and index scores used in the present study had been proven to be reliable. In their study, the quantification of progress towards SDGs at national and regional levels was only based on the SDG index score, which refers to the mean index score (MIS) in the present study. We further calculated the SDG evenness score (ES) based on all 17 SDG scores, then computed the sustainable development score (SDS) that is an integration of both ES and MIS to quantify the progress towards all 17 SDGs at national and regional levels in China. Additionally, we compared the performance of sustainable development between economically developing regions and economically developed regions, which were defined by the average GDP per capita of each province from 2000 to 2015 [2]. Provinces with the highest 10 and the lowest 10 GDP values were considered as economically developed provinces and developing provinces, respectively.

### Calculations for evenness score and sustainable development score

The generally used index to assess the progress towards sustainable development is an aggregate score using the arithmetic mean of all 17 SDG scores [2,4], which might be biased and over-optimistic when large variances exist among 17 SDG scores. In such a case, the achievement of SDGs mainly depends on the poorly achieved SDGs rather than the almost accomplished ones, whereas the mean value of 17 SDGs could be high despite the existence of those poorly achieved SDGs. For instance, a region with an SDG score of 100 (best performance) for half of its SDGs and 0 (worst performance) for the rest still has an SDG index score of 50; however, it is hard to say that this region is halfway to achieving SDGs, given that its efforts might mainly focus on a few selected SDGs. Therefore, the evenness among all 17 SDGs needs to be adopted to diminish the potential over-estimation of sustainable development performance by using the SDG index score only.

An improved radar chart method [20,37] was used to compute the SDG evenness score from the SDG index score, because (i) it could visualize the SDG index score (by its area), the evenness among all 17 SDGs (by its perimeter) and the score of each SDG (by the radius of each SDG) (Fig. S1); (ii) it provided a visualized comparison of the overall status of all SDGs and scores for each SDG over time or across space (Fig. S1); (iii) the calculation of evenness score was not affected by the order of the 17 SDGs [37]. The score of each SDG at the national level or a given province in a certain year was used as the radius of each sector; therefore, the radar chart was formed by 17 sectors corresponding to the 17 SDGs. The area (*S*) and perimeter (*L*) of the radar chart are expressed as follows (Fig. S5):

(1) \begin{equation*} {S_i} = \mathop \sum \limits_{j = 1}^n {S_{\!j}} = \mathop \sum \limits_{j = 1}^n \pi {f_{\!j}}{r_{\!j}}^{\!\!2},\ \ j = 1,2, \ldots ,n,\end{equation*}

(2) \begin{eqnarray*} {L_i} &=& \mathop \sum \limits_{j = 1}^n {L_{\!j}} = 2\left| {{r_{\rm max}} - {r_{\rm min}}} \right| \nonumber\\ && +\, \mathop \sum \limits_{j = 1}^n 2\pi {f_{\!j}}{r_{\!j}},\ \ j = 1,2, \ldots ,n. \end{eqnarray*}

In the calculation of evenness among 17 SDGs at national and regional levels, *n* represents the number of SDGs which is 17, *r*_max_ and *r*_min_ represent the maximum and minimum among 17 SDG scores, respectively, and *r_j_* refers to the score of the *j^th^* SDG. In the calculation of the evenness of a given SDG among 31 provinces, *n* represents the number of provinces, *r*_max_ and *r*_min_ represent the maximum and minimum of the corresponding SDG score among all 31 provinces, respectively, and *r_j_* refers to the corresponding SDG score of the *j^th^* province. *f_j_* stands for the weight of the *j^th^* SDG, which is 1/17 for all SDGs because there is no reason to give one SDG greater importance than another [3,4]. Notably, the doubled value of the difference between *r*_max_ and *r*_min_ refers to the part of perimeter other than the total length of all arcs (the total length of all lines between two adjacent arcs; Fig. S5). Evenness score refers to the ratio between the total area of the radar chart formed by the 17 SDGs and the area of a circle with the same perimeter (the evenest distribution of all SDGs with the same perimeter), which is calculated with *S_i_* and *L_i_*based on equation (3). It is multiplied by 100 to be comparable with the SDG index score (0–100). The area of the radar chart with a fixed perimeter reaches its largest (100) when it is a circle, and decreases with increasing unevenness among all radii (referring to scores of all 17 SDGs in the present study) [37]. Therefore, the ES is the highest when all SDGs have the same score.

(3) \begin{eqnarray*} {\text {ES}} = {S_i}/\left[ {\pi {{\left( {{L_i}/2\pi } \right)}^2}} \right] \times 100 = 400\pi {S_i}/{L_i}^{\!\!\!2}.\nonumber\\ \end{eqnarray*}

The geometric mean of the evenness score and the mean index score were used to represent the SDS, which diminishes the potential overestimation of sustainable development performance by using the mean index score only when large variances among 17 SDG scores exist. All these scores range from 0 (worst performance) to 100 (best performance). As the upper/lower boundary selection and normalization of each SDG score were based on acceptable approaches worldwide, namely, the SDG Index and Dashboards Report [4], our results can be comparable to the existing SDGs assessment results following the same approaches.

Overall, we annually calculated evenness scores and sustainable development scores based on China's 17 SDG scores from 2000 to 2015, yielding 16 evenness scores and 16 sustainable development scores at the national level. At the provincial level, evenness scores and sustainable development scores for each province were calculated from its 17 SDG scores in the years of 2000, 2005, 2010 and 2015, separately, yielding four evenness scores and four sustainable development scores per province.

### Definitions for the developing pathway and the effective development score

**Figure 6.**
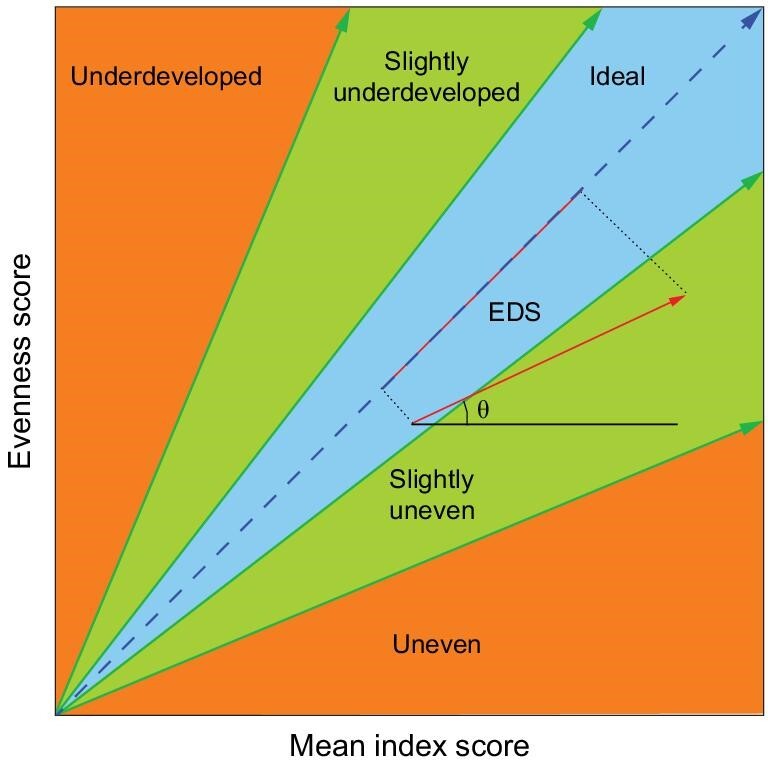
Sketch presenting the definition of the developing pathway and the effective development score (EDS). θ is the angle between a given vector and the x-axis.

By plotting the pairwise mean index score (x) and evenness score (y) of China or a certain province in 2000 and 2015, we could visualize the developing pathway from 2000 to 2015 by constructing a vector starting with the paired values in 2000 and ending with the paired values in 2015. The perfect pathway was defined as the vector with a slope of one, indicating a simultaneous improvement in all 17 SDGs. As a decreasing trend was found neither in the mean index score nor in the evenness score, the worst scenario is that only the mean index score or the evenness score increases over time, limiting the range of the angle (θ) between the vector and x-axis from 0° to 90°. Therefore, we divided the 90-degree space centered on the perfect pathway into two 45-degree ranges (Fig. 6). The central 45° was considered as a sound pathway, while it was further divided into three subgroups, namely, slightly uneven (22.5° < θ < 37.5°), relatively ideal (37.5° < θ < 52.5°) and slightly underdeveloped (52.5° < θ < 67.5°). The other two ends were classified as uneven (0° < θ < 22.5°) and underdeveloped (67.5° < θ < 90°). Furthermore, considering both the change of mean index score and evenness score, we developed an index called the effective development score (EDS) to quantify the progress towards SDGs from 2000 to 2015. The projected length (red line in Fig. 6) of a given pathway (red vector) on the perfect pathway was used to represent EDS, which helps to diminish the over-estimation of progress towards SDGs over time when the achievements are majorly embodied in increasing mean index score (improvements only shown in a few SDGs) or increasing evenness score (increasing scores for the poorly achieved SDGs while decreasing scores for the better accomplished SDGs).

### Assessment of the relative developing status of 31 provinces in 2015

To explore the different stages towards SDGs among 31 provinces in China, we plotted the mean index score (x) of each province against its evenness score (y) in 2015. Then, we used the K-mean method to divide the 31 provinces into two groups (relatively high and relatively low) based on the mean index score (MIS) or the evenness score (ES), respectively (namely, the relatively high MIS group and the relatively low MIS group, based on MIS; the relatively high ES group and the relatively low ES group, based on ES). Furthermore, all 31 provinces could be divided into four categories in terms of the relative developing status, i.e. relatively sustainably developed (provinces with relatively high MIS and ES), relatively underdeveloped (provinces with relatively low MIS and high ES), relatively uneven (provinces with relatively high MIS and low ES) and relatively underdeveloped and uneven (provinces with relatively low MIS and ES). The reader should notice that the four categories were based on the relative developing status among 31 provinces in 2015, instead of their current SDG status.

## Supplementary Material

nwaa238_Supplemental_File
