# Supplementary material for: Evenness is important in assessing progress towards sustainable development goals
Source: Natl Sci Rev. 2020 Sep 28;8(8):nwaa238. doi: 10.1093/nsr/nwaa238 (PMC8363329; doi:10.1093/nsr/nwaa238)
Supplement: nwaa238_Supplemental_File [file nwaa238_supplemental_file.docx]

Supplementary Materials for

Evenness is important in assessing progress towards sustainable development goals

Yali Liu†, Jianqing Du†, Yanfen Wang^*^, Xiaoyong Cui, Jichang Dong, Yanbin Hao, Kai Xue, Hongbo Duan, Anquan Xia, Yi Hu, Zhi Dong, Bingfang Wu, Xinquan Zhao, Bojie Fu

^*^Correspondence to: yfwang@ucas.ac.cn

† These authors contributed equally to this work

**This word file includes:**

Figures S1-S5

Tables S1-S6





Figure S1.

China’s 17 SDGs index scores in 2000, 2008, and 2015. The radar chart starts from the north with the SDG with the highest index score in 2015. It visualizes the changing index score of each SDG from 2000 to 2008, and from 2008 to 2015.





Figure S2.

Changes in the evenness score (ES) of each SDG across 31 provinces between 2000 and 2015. Positive values represent increasing ES from 2000 to 2015.





Figure S3.

Relative sustainable development statuses at national and regional levels in 2000, 2005, 2010, and 2015. As 2015 is the midway between the start of Millennium Development (2000) and the deadline for achieving SDGs (2030), a score of 50 is set as the threshold for both ES and MIS to distinguish between relatively good and laggard status. Refer to Fig. 1, the four quadrants formed by the two gray lines represent relatively ideal status, underdeveloped status, underdeveloped and uneven status, and uneven status, respectively. Dots represent 31 provinces, pentagrams stand for the national status.





Figure S4.

Comparison of evenness scores and sustainable development scores for different groups of provinces in China. (A) evenness scores; (B) sustainable development scores. Bottom 5 and top 5 stand for the bottom five developing (poorest) provinces and the top five developed (richest) provinces in China. Developing and developed represent the bottom ten developing (poorest) provinces and the top ten developed (richest) provinces in China. The histogram with error bars presents the mean value ± standard deviation (SD). Letters a, b, c, and d visualize the significant differences at *p* < 0.05 (Repeated ANOVA, Least Significant Difference test). For the detailed definition of each category, see Methods.


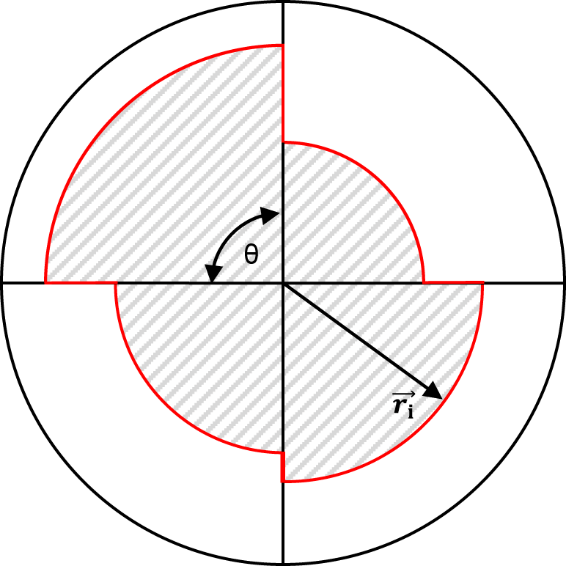


Figure S5.

Outline of the radar chart method. *r_j_* stands for the score of the jth SDG. θ represents the weight of each SDG which refers to *f_j_* in equation (1) and (2), and is set up to 1/17 for all SDGs in the present study. The sum of the area of each sector refers to *S_i_* in equation (1). The red line presents the total perimeter of all sectors which refers to *L_i_* in equation (2).

Table S1.

Mean index score, evenness score, and sustainable development score for 31 provinces over time

| **Regions** | **Provinces** | **Mean index score** | | | | **Evenness score** | | | | **Sustainable development score** | | | |
| --- | --- | --- | --- | --- | --- | --- | --- | --- | --- | --- | --- | --- | --- |
|  |  | **2000** | **2005** | **2010** | **2015** | **2000** | **2005** | **2010** | **2015** | **2000** | **2005** | **2010** | **2015** |
| Northeast China | Heilongjiang | 40.86 | 45.46 | 52.59 | 54.88 | 53.40 | 52.94 | 62.36 | 64.65 | 46.71 | 49.06 | 57.27 | 59.56 |
|  | Jilin | 41.16 | 47.28 | 49.71 | 57.49 | 54.44 | 55.81 | 66.99 | 64.28 | 47.34 | 51.37 | 57.71 | 60.79 |
|  | Liaoning | 41.92 | 48.47 | 52.92 | 55.06 | 56.91 | 60.14 | 68.28 | 66.93 | 48.84 | 53.99 | 60.12 | 60.70 |
| Northwest China | Xinjiang | 39.97 | 41.74 | 47.36 | 49.16 | 57.23 | 57.08 | 65.58 | 69.77 | 47.83 | 48.81 | 55.73 | 58.56 |
|  | Gansu | 35.63 | 42.71 | 42.49 | 50.31 | 46.35 | 53.69 | 65.66 | 67.77 | 40.64 | 47.88 | 52.82 | 58.39 |
|  | Qinghai | 42.12 | 39.38 | 46.93 | 49.71 | 55.41 | 61.63 | 60.37 | 68.17 | 48.31 | 49.26 | 53.23 | 58.21 |
|  | Ningxia | 31.45 | 38.06 | 43.11 | 47.73 | 56.20 | 58.64 | 61.01 | 62.63 | 42.04 | 47.24 | 51.28 | 54.67 |
|  | Shaanxi | 42.41 | 45.07 | 49.68 | 54.48 | 54.09 | 52.26 | 62.65 | 69.19 | 47.89 | 48.53 | 55.78 | 61.40 |
| North China | Inner Mongolia | 35.13 | 42.06 | 45.41 | 48.82 | 49.89 | 56.11 | 64.71 | 64.41 | 41.86 | 48.58 | 54.21 | 56.08 |
|  | Beijing | 52.50 | 57.61 | 63.92 | 66.12 | 60.65 | 59.41 | 59.42 | 62.94 | 56.43 | 58.50 | 61.63 | 64.51 |
|  | Tianjin | 46.52 | 53.12 | 54.71 | 57.30 | 54.35 | 62.80 | 66.11 | 59.77 | 50.28 | 57.76 | 60.14 | 58.52 |
|  | Hebei | 41.07 | 42.40 | 46.65 | 49.93 | 52.50 | 52.60 | 64.06 | 64.19 | 46.44 | 47.22 | 54.66 | 56.61 |
|  | Shanxi | 37.29 | 40.12 | 44.33 | 46.98 | 61.47 | 61.51 | 62.83 | 63.70 | 47.88 | 49.68 | 52.78 | 54.70 |
| Central South China | Henan | 42.90 | 45.81 | 51.18 | 54.44 | 50.50 | 51.77 | 58.88 | 63.74 | 46.54 | 48.70 | 54.90 | 58.91 |
|  | Hubei | 41.93 | 43.18 | 51.29 | 57.19 | 49.20 | 48.10 | 65.52 | 70.09 | 45.42 | 45.57 | 57.97 | 63.32 |
|  | Hunan | 45.59 | 42.53 | 51.55 | 56.08 | 55.10 | 53.34 | 60.70 | 64.80 | 50.12 | 47.63 | 55.94 | 60.28 |
|  | Guangdong | 50.20 | 51.15 | 51.26 | 56.36 | 54.83 | 58.53 | 59.82 | 65.75 | 52.46 | 54.71 | 55.38 | 60.88 |
|  | Guangxi | 41.12 | 43.87 | 47.26 | 55.88 | 51.27 | 56.45 | 66.37 | 65.84 | 45.92 | 49.76 | 56.01 | 60.66 |
|  | Hainan | 42.95 | 43.48 | 45.24 | 54.88 | 50.88 | 55.67 | 50.29 | 61.44 | 46.75 | 49.20 | 47.70 | 58.07 |
| East China | Shanghai | 54.11 | 53.67 | 57.98 | 60.12 | 60.08 | 62.99 | 64.85 | 61.84 | 57.01 | 58.14 | 61.32 | 60.98 |
|  | Jiangsu | 46.01 | 48.41 | 50.85 | 55.26 | 55.80 | 58.94 | 63.48 | 62.92 | 50.67 | 53.41 | 56.81 | 58.96 |
|  | Zhejiang | 44.78 | 47.09 | 54.68 | 57.77 | 49.96 | 58.54 | 62.69 | 66.78 | 47.30 | 52.51 | 58.55 | 62.11 |
|  | Anhui | 40.46 | 43.36 | 51.47 | 53.97 | 51.02 | 51.97 | 62.55 | 68.45 | 45.44 | 47.47 | 56.74 | 60.78 |
|  | Jiangxi | 39.18 | 43.07 | 46.56 | 55.45 | 50.51 | 53.85 | 61.01 | 67.84 | 44.49 | 48.16 | 53.30 | 61.33 |
|  | Shandong | 46.35 | 47.38 | 50.86 | 54.14 | 51.36 | 55.88 | 64.45 | 63.77 | 48.79 | 51.45 | 57.25 | 58.76 |
|  | Fujian | 44.21 | 44.24 | 50.55 | 59.16 | 52.41 | 58.09 | 51.96 | 65.34 | 48.13 | 50.69 | 51.25 | 62.18 |
| Southwest China | Tibet | 37.92 | 41.03 | 44.75 | 51.64 | 53.56 | 53.77 | 60.75 | 63.43 | 45.07 | 46.97 | 52.14 | 57.23 |
|  | Sichuan | 43.93 | 47.71 | 51.43 | 58.84 | 55.25 | 54.72 | 66.31 | 68.64 | 49.27 | 51.10 | 58.40 | 63.55 |
|  | Chongqing | 41.01 | 43.52 | 52.64 | 60.54 | 57.06 | 64.23 | 72.22 | 70.89 | 48.37 | 52.87 | 61.66 | 65.51 |
|  | Guizhou | 36.79 | 41.89 | 45.59 | 55.76 | 54.70 | 55.32 | 64.42 | 69.12 | 44.86 | 48.14 | 54.20 | 62.08 |
|  | Yunnan | 42.20 | 45.38 | 49.00 | 56.96 | 56.96 | 53.94 | 62.42 | 65.12 | 49.03 | 49.48 | 55.30 | 60.90 |

Table S2.

Evenness score of each SDG among 31 provinces over time

| Year | SDG 1 | SDG 2 | SDG 3 | SDG 4 | SDG 5 | SDG 6 | SDG 7 | SDG 8 | SDG 9 | SDG 10 | SDG 11 | SDG 12 | SDG 13 | SDG 14 | SDG 15 | SDG 16 | SDG 17 |
| --- | --- | --- | --- | --- | --- | --- | --- | --- | --- | --- | --- | --- | --- | --- | --- | --- | --- |
| **2000** | 58.00 | 66.06 | 70.34 | 65.94 | 64.17 | 58.98 | 51.43 | 48.89 | 45.00 | 47.86 | 66.45 | 79.80 | 72.20 | 53.21 | 70.11 | 67.32 | 41.45 |
| **2005** | 56.85 | 71.17 | 67.24 | 64.73 | 71.77 | 61.14 | 57.64 | 49.51 | 46.58 | 47.71 | 66.50 | 75.76 | 73.84 | 54.56 | 63.24 | 68.69 | 48.51 |
| **2010** | 59.68 | 70.63 | 74.36 | 66.75 | 77.90 | 65.13 | 58.00 | 60.47 | 53.91 | 60.31 | 64.35 | 73.83 | 74.64 | 36.06 | 61.52 | 62.35 | 57.75 |
| **2015** | 65.17 | 73.06 | 82.09 | 70.35 | 70.67 | 62.02 | 58.19 | 66.08 | 60.04 | 72.36 | 68.64 | 66.19 | 77.74 | 61.15 | 64.04 | 68.64 | 62.14 |
| **Change** | 7.17 | 7.00 | 11.75 | 4.41 | 6.50 | 3.04 | 6.76 | 17.18 | 15.04 | 24.50 | 2.20 | -13.62 | 5.54 | 7.94 | -6.07 | 1.32 | 20.69 |

Note: Change represents the change from 2000 to 2015, positive values suggest an increase in evenness score.

Table S3.

The developing pathways for 31 provinces from 2000 to 2015

| **Regions** | **Uneven (θ)** | **Slightly uneven (θ)** | **Slightly underdeveloped (θ)** | **Relatively ideal (θ)** |
| --- | --- | --- | --- | --- |
| Northeast |  | Jilin (31.07°) |  | Heilongjiang (38.78°) |
|  |  | Liaoning (37.30°) |  |  |
| Northwest | Ningxia (21.56°) |  | Xinjiang (53.78) | Shaanxi (51.35°) |
|  |  |  | Gansu (55.56°) |  |
|  |  |  | Qinghai (59.26°) |  |
| North | Beijing (9.54°) | Tianjin (26.70°) | Hebei (52.83°) | Inner Mongolia (46.67°) |
|  | Shanxi (12.99°) |  |  |  |
| Central South |  |  | Hubei (53.85°)  Guangdong (60.56°) | Hainan (41.53°) |
|  |  |  |  | Hunan (42.80°) |
|  |  |  |  | Guangxi (44.64°) |
|  |  |  |  | Henan (48.94°) |
| East | Shanghai (16.34°) |  | Shandong (57.91°) | Jiangsu (37.57°) |
|  |  |  |  | Fujian (40.84°) |
|  |  |  |  | Jiangxi (46.80°) |
|  |  |  |  | Anhui (52.22°) |
|  |  |  |  | Zhejiang (52.32) |
| Southwest |  | Yunnan (28.94°) |  | Sichuan (41.93°) |
|  |  | Chongqing (35.32°) |  |  |
|  |  | Tibet (35.73°) |  |  |
|  |  | Guizhou (37.24°) |  |  |

Table S4.

Corresponding provinces of Fig. 4B and their effective development scores

| Northeast | Jilin  (18.50) | Heilongjiang  (17.87) | Liaoning  (16.37) |  |  |  |  |
| --- | --- | --- | --- | --- | --- | --- | --- |
| Northwest | Gansu  (25.53) | Shaanxi  (19.21) | Ningxia  (16.06) | Xinjiang  (15.37) | Qinghai  (14.39) |  |  |
| North | Inner Mongolia  (19.95) | Hebei  (14.53) | Tianjin  (11.45) | Beijing  (11.25) | Shanxi  (8.42) |  |  |
| Southwest | Guizhou  (23.61) | Chongqing  (23.59) | Sichuan  (20.00) | Tibet  (16.68) | Yunnan  (16.21) |  |  |
| Central South | Hubei  (25.57) | Guangxi  (20.74) | Henan  (17.52) | Hainan  (15.90) | Hunan  (14.27) | Guangdong  (12.08) |  |
| East | Jiangxi  (23.75) | Anhui  (21.87) | Zhejiang  (21.07) | Fujian  (19.72) | Shandong  (14.28) | Jiangsu  (11.58) | Shanghai  (5.50) |

Table S5.

Evenness score and sustainable development score between developing and developed regions over time

| **Regions** | **Evenness score** | | | | **Sustainable development score** | | | |
| --- | --- | --- | --- | --- | --- | --- | --- | --- |
|  | **2000** | **2005** | **2010** | **2015** | **2000** | **2005** | **2010** | **2015** |
| Bottom 5 developing provinces | 52.57 | 54.63 | 63.92 | 66.26 | 45.10 | 48.45 | 54.09 | 59.85 |
| Top 5 developed provinces | 56.17 | 60.54 | 63.31 | 62.85 | 52.34 | 56.07 | 59.69 | 61.02 |
| Developing provinces | 53.16 | 54.70 | 63.12 | 66.36 | 45.91 | 48.73 | 54.66 | 59.85 |
| Developed provinces | 54.62 | 59.14 | 62.58 | 64.04 | 50.18 | 53.98 | 57.67 | 60.37 |

Note: Bottom 5 developing provinces are Guizhou, Gansu, Yunnan, Tibet, Guangxi; Other developing provinces include Anhui, Jiangxi, Sichuan, Shanxi, and Henan; Top 5 developed provinces are Zhejiang, Jiangsu, Beijing, Shanghai, Tianjin; Other developed provinces include Fujian, Shandong, Guangdong, Liaoning, and Inner Mongolia.

Table S6.

Regional differences in developing status among 31 provinces in 2015

| **Regions** | **Underdeveloped & uneven** | **Underdeveloped** | **Uneven** | **Relatively sustainably developed** |
| --- | --- | --- | --- | --- |
| Northeast |  |  | Heilongjiang  Jilin | Liaoning |
| Southwest | Tibet |  | Yunnan | Chongqing  Guizhou  Sichuan |
| North | Shanxi  Inner Mongolia  Hebei |  | Tianjin  Beijing |  |
| Central South |  |  | Guangxi | Hubei |
|  |  |  | Guangdong |  |
|  |  |  | Hunan |  |
|  |  |  | Henan |  |
|  |  |  | Hainan |  |
| East |  |  | Fujian | Anhui  Jiangxi  Zhejiang |
|  |  |  | Shandong |  |
|  |  |  | Jiangsu |  |
|  |  |  | Shanghai |  |
| Northwest | Ningxia | Xinjiang |  | Shaanxi |
|  |  | Qinghai |  |  |
|  |  | Gansu |  |  |
